# Primary Squamous Cell Carcinoma of the Stomach: A Case Report

**DOI:** 10.7759/cureus.54188

**Published:** 2024-02-14

**Authors:** Ygor R Fernandes, Ketlin B Morais, Ana Carolina Campos, Rodrigo S Machado

**Affiliations:** 1 Center of Digestive Endoscopy of University of São Paulo, Universidade Federal de São Paulo/Escola Paulista de Medicina/Hospital São Paulo, São Paulo, BRA; 2 Center of Digestive Endoscopy, Federal University of São Paulo, São Paulo, BRA; 3 Center of Digestive Endoscopy, Hospital São Paulo/Federal University of São Paulo, São Paulo, BRA; 4 Division of Pediatric Gastroenterology, Department of Pediatrics, Center of Digestive Endoscopy, Hospital São Paulo/Federal University of São Paulo, São Paulo, BRA

**Keywords:** gastric tumor, tumor, squamous cell carcinoma, carcinoma, stomach

## Abstract

Gastric squamous cell carcinoma (SCC) is a rare and puzzling entity that challenges conventional paradigms of gastric malignancies, especially in young adults. This case report presents a 22-year-old male with invasive SCC of the stomach, emphasizing the rarity of such occurrences and their diagnostic challenges. The literature review underscores the scarcity of information on gastric SCC, necessitating a critical examination of its clinical implications, etiological factors, and optimal management. The patient’s complex medical history, diagnostic journey, and treatment course are detailed, highlighting the importance of multidisciplinary collaboration and advanced diagnostic techniques. Immunohistochemistry is a crucial tool for precise tumor characterization, and the absence of established risk factors emphasizes the enigmatic nature of gastric SCC. This case report contributes to the understanding of gastric SCC, prompting further research into its unique features, etiology, and therapeutic strategies in the context of gastric cancer.

## Introduction

Gastric carcinoma, predominantly of the adenocarcinoma subtype, is a well-established malignancy. It constitutes approximately 95% of all gastric malignancies, with a significant global prevalence, becoming the fifth most prevalent, with 5.7% of all new cases attributable to the disease [1]. The global burden of gastric cancer is substantial, accounting for a significant proportion of cancer-related morbidity and mortality, and is the leading oncological cause of death in 10 nations worldwide [1]. In contrast, squamous cell carcinoma (SCC) is a rare type of tumor in the stomach, comprising only a small fraction of reported cases with a worldwide incidence of 0.04% to 0.07% of all gastric cancers and with fewer than 100 cases reported in the literature to date [2]. It occurs mostly among men, and the male-to-female ratio is 5 to 1 [2]. Here, we report the case of a 22-year-old male presenting with an uncommon diagnosis of invasive SCC of the stomach, an anomaly that challenges the conventional paradigm of gastric malignancies.

The rarity of gastric SCC prompts a critical examination of its etiological factors, clinical manifestations, and optimal management strategies. There is sparse data in the literature on the molecular mechanisms underpinning gastric SCC, and its association with specific risk factors in young adults is poorly defined [2,3]. This case report endeavors to contribute to this knowledge gap by presenting a detailed account of the diagnostic journey, including clinical, radiological, and histopathological aspects. Gastric SCC is scarcely prevalent in individuals under the age of 30, rendering our case noteworthy within the context of existing literature, as it most commonly manifests after the sixth decade of life [1-3].

This case underscores the importance of multidisciplinary collaboration and advanced diagnostic techniques in navigating the complexities of rare gastric malignancies. Through the lens of this unique case, correlating gastric SCC with immunodeficiency and celiac disease, we aim to not only enhance the understanding of invasive SCC of the stomach but also stimulate further investigations into its pathogenesis and therapeutic modalities.

## Case presentation

A 22-year-old male, a non-smoker, was referred to the endoscopy unit with involuntary weight loss and abdominal pain. He had been followed up in the university hospital for over 20 years due to a diagnosis of celiac disease when he was one year old. Additionally, he had been diagnosed with Evans syndrome, IgA deficiency, and recurrent pneumonia and had been undergoing immunology follow-up with gammaglobulin therapy for 10 years. His follow-up during childhood was irregular, with difficult compliance to a gluten-free diet. Eventually, he was lost to follow-up after the age of 11, with just some sporadic appointments. In one of these, an upper gastrointestinal endoscopy revealed atrophic gastritis and intestinal metaplasia when he was only 18 years old.

In adulthood, he sought to resume medical treatment in our institution. However, over the last seven months, the patient experienced significant and involuntary weight loss (10 kg) accompanied by abdominal pain related to meals. He was admitted to the hospital for further evaluation and management of severe malnutrition.

**Figure 1:**
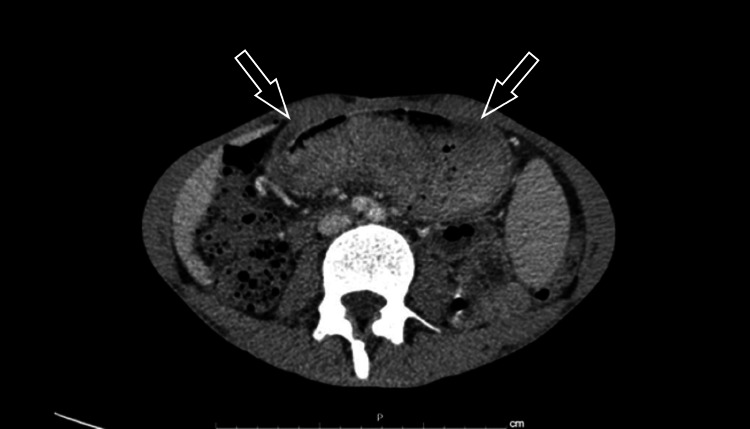
CT reveals a large exophytic mass in the gastric body and antrum with infiltration of the adjacent fat.

The patient’s physical examination was unremarkable. Laboratory results showed macrocytic anemia (hemoglobin: 9.8 g/dL), hypocalcemia (8.1 mg/dL), hyponatremia (132 mEq/L), and normal carcinoembryonic antigen (CEA) (2 ng/mL). Contrast-enhanced CT of the abdomen and pelvis showed asymmetrical parietal thickening of the gastric wall, with an irregular area in the great curvature, measuring up to 4.5 cm, suspicious for a tumor (Figure 1).

**Figure 2:**
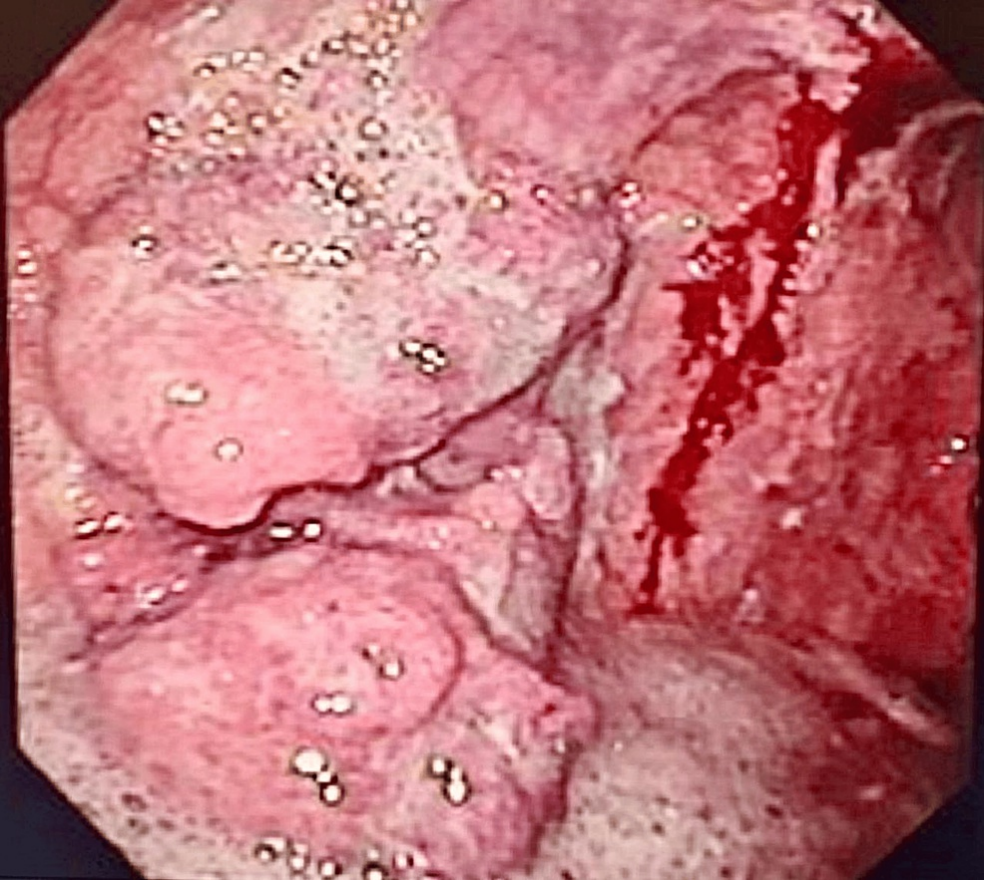
Lesion in the lesser curvature.

**Figure 3:**
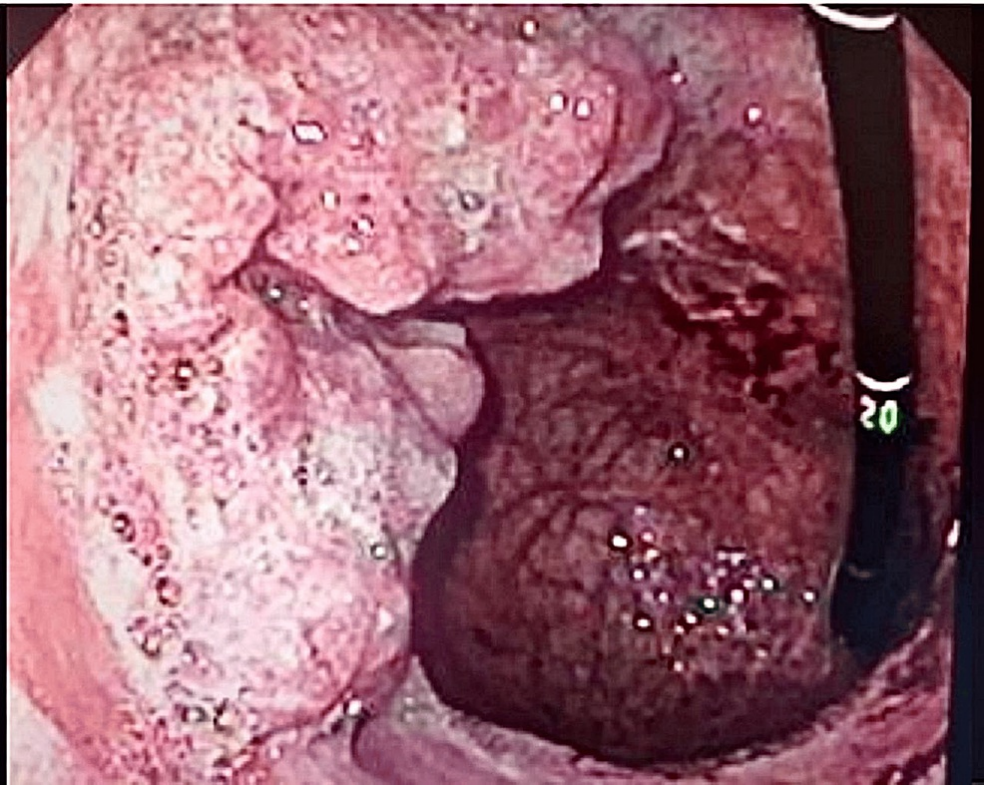
Lesion in the greater curvature at the gastric antrum.

Upper digestive endoscopy showed two lesions, one infiltrative protruded lesion along the lesser curvature of the body (Figure 2) with irregular contours, friable, occupying more than 50% of the organ’s circumference. The other mass was infiltrative with ulceration along the greater curvature at the gastric antrum, with imprecise contours (Figure 3).

**Figure 4:**
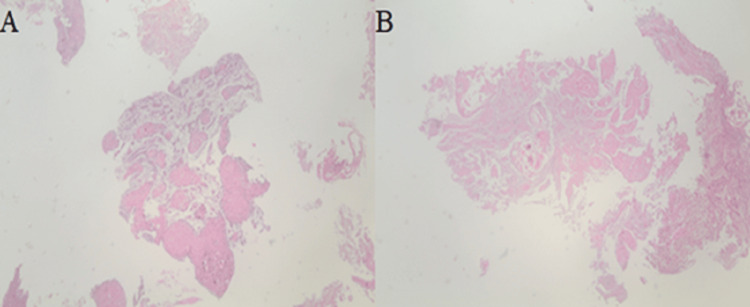
(A) Gastric mucosa substituted for a proliferation of keratinized cells infiltrating the muscle layer (hematoxylin and eosin, original magnification ×40). (B) The tumoral area composed of cells with ample eosinophilic cytoplasm with occasional keratinization and showing necrosis (hematoxylin and eosin, original magnification ×40).

**Figure 5:**
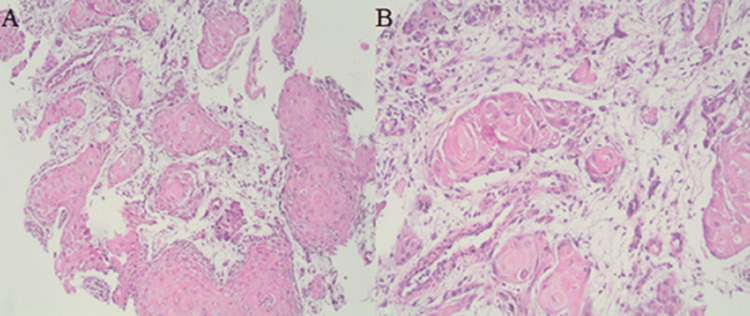
(A) Gastric mucosa substituted for a proliferation of keratinized cells infiltrating the muscle layer – well-differentiated squamous cell carcinoma (hematoxylin and eosin, original magnification ×100). (B) Gastric mucosa substituted for a proliferation of keratinized cells infiltrating the muscle layer - well-differentiated squamous cell carcinoma (hematoxylin and eosin, original magnification ×200).

Multiple biopsies were taken from both lesions using standard biopsy forceps, which later revealed invasive SCC on histopathological examination (Figures 4, 5).

The patient’s general clinical condition worsened and he was planned for emergency surgery. Distal subtotal gastrectomy with standard D2 lymphadenectomy and Roux-en-Y reconstruction was performed. The surgical specimen showed an ulcer lesion measuring around 9 x 8 cm with macroscopic infiltration up to the adipose tissue, with free margins and no lymph node involvement. The final pathological study confirmed a moderately differentiated SCC (T4aN0M0).

## Discussion

The occurrence of invasive SCC in the stomach, as illustrated in this case of a 22-year-old male, is a notable rarity in the context of gastric malignancies. Patients diagnosed with gastric SCC may present with a range of clinical symptoms [4]. These symptoms include abdominal pain, dysphagia, nausea and vomiting, melena or hematochezia, hematemesis, and weight loss [4]. It is important to note that many of these symptoms overlap with those of other gastric tumors, making them clinically indistinguishable [4,5].

The aggressive nature of gastric SCC, as evidenced by the locally advanced stage at diagnosis and the limited therapeutic options, resonates with existing literature [3-5]. The anatomical location of the tumor, involving both the antrum and the great curvature, aligns with reports of a predilection for the distal stomach [4,5]. This aggressive behavior and distinctive location underscore the need for heightened clinical awareness [5].

Immunohistochemistry (IHC) is a crucial tool in confirming the diagnosis and characterizing the tumor [6]. It categorizes patients to ensure appropriate and specific treatment, as well as helps identify tumors at higher risk of recurrence and lethal outcomes [6,7]. The limitations of established immunohistochemical markers for gastric SCC compared to SCC in other organs highlight the need for further research to delineate specific diagnostic immunohistochemical signatures [7,8]. The recommended IHC panel for diagnosing and predicting prognosis involves assessing the expression of various markers, including CK5, CK7, CK20, TTF1, p63, chromogranin, synaptophysin, CEA, and cancer antigen 19-9 [7,8].

Despite the importance of IHC as a crucial tool in confirming diagnoses and characterizing tumors, its application in our specific case was hindered by unavailability.

Evans syndrome is a rare autoimmune disorder characterized by the coexistence of autoimmune hemolytic anemia and immune thrombocytopenia [9]. The treatment approach for Evans syndrome typically involves a combination of immunosuppressive drugs aimed at modulating the overactive immune response [9]. Achieving effective control of hemolytic anemia is critical to ensuring a positive overall prognosis for individuals with this syndrome [9].

Fortunately, the prognosis for Evans syndrome is generally favorable once hemolytic anemia is successfully controlled [9,10]. However, the association of the syndrome with malignancies remains a topic of ongoing research, with some cases showing a potential association with certain cancers, but nothing related to gastric cancer [10]. Further studies are needed to elucidate the exact nature of this association and its implications for individuals with Evans syndrome.

In contrast, celiac disease, another autoimmune disorder, shows a clear association with lymphoproliferative gastrointestinal malignancies [11]. Notably, celiac disease is not commonly associated with tumors of epithelial origin [11]. This underscores the importance of understanding the unique associations and patterns of malignancy in different autoimmune diseases, which may contribute to more targeted and effective management strategies.

In exploring potential etiological factors and known risk elements, the scarcity of information in the literature becomes apparent. Gastric SCC is seldom associated with conventional risk factors for gastric adenocarcinomas, such as *Helicobacter pylori* infection, dietary factors, and tabagism [12]. The lack of established risk factors for gastric SCC emphasizes the puzzling nature of its pathogenesis, warranting further investigation into its unique etiological contributors. The rarity of gastric SCC, particularly in young adults, further emphasizes the need for a more extensive body of literature to guide clinicians in navigating diagnostic and therapeutic dilemmas.

The overall survival rate ranges from seven months to eight years [13]. Although the primary treatment is surgery, it seems that adjuvant chemotherapy (5-fluorouracil, leucovorin, cisplatin, and etoposide) can enhance survival rates [13]; however, there is insufficient evidence to confirm this claim. Therefore, the effectiveness of adjuvant chemotherapy and radiotherapy (cone-beam CT) in achieving long-term survival remains uncertain, as there is only a single prior study that showed the efficacy of chemotherapy in combating this tumor [13,14].

## Conclusions

This case report not only sheds light on the distinctive clinical and pathological features of gastric SCC but also underscores the pressing need for continued research to elucidate its etiological factors, molecular underpinnings, and optimal therapeutic strategies. The scarcity of established risk factors and the aggressive behavior of this malignancy underscore the importance of this contribution to the evolving landscape of gastric cancers.
